# P91 Antimicrobial activity of ceftolozane/tazobactam and comparators against clinical Gram-negative bacilli collected in the UK: results from the Study for Monitoring Antimicrobial Resistance Trends (SMART) 2020–24

**DOI:** 10.1093/jacamr/dlag102.097

**Published:** 2026-06-26

**Authors:** M Wise, M Allen, F Siddiqui, K Young, M Motyl, D Sahm

**Affiliations:** IHMA, Schaumburg, IL, USA; MSD (UK) Limited, London, UK; Merck & Co. Inc., Rahway, NJ, USA; Merck & Co. Inc., Rahway, NJ, USA; Merck & Co. Inc., Rahway, NJ, USA; IHMA, Schaumburg, IL, USA

## Abstract

**Background:**

Ceftolozane/tazobactam (C/T) is an antipseudomonal cephalosporin combined with a β-lactamase inhibitor approved by the MHRA, EMA and FDA for complicated urinary tract and intraabdominal infections in adults and children, and hospital-acquired/ventilator-associated bacterial pneumonia in adults. We evaluated the activity of C/T against isolates of Enterobacterales and *Pseudomonas aeruginosa* collected in the UK as part of the global SMART surveillance programme.

**Methods:**

In 2020-2024, 6 clinical laboratories in the UK each collected up to 250 consecutive, aerobic or facultative, Gram-negative pathogens per year from patients with bloodstream, intraabdominal, urinary tract, and lower respiratory tract infections. MICs were determined using the reference broth microdilution methods and interpreted with 2025 EUCAST breakpoints. An ESBL-positive, non-CRE (non-carbapenem-resistant Enterobacterales) phenotype was defined for isolates of *Escherichia coli* and *Klebsiella pneumoniae* as those testing with a ceftriaxone MIC ≥2 mg/L and an ertapenem MIC ≤0.5 mg/L. For Enterobacterales, an MDR phenotype was defined as resistance (by EUCAST criteria) to ≥3 sentinel agents (amikacin, cefepime, colistin, levofloxacin, meropenem and piperacillin/tazobactam). For *P. aeruginosa*, MDR was defined as an isolate testing as resistant (EUCAST) to ≥3 sentinel drugs: amikacin, aztreonam, cefepime, colistin, meropenem, levofloxacin and piperacillin/tazobactam. Difficult-to-treat resistant (DTR) *P. aeruginosa* was defined for isolates testing resistant by EUCAST to all β-lactams (including aztreonam, cefepime, ceftazidime, imipenem, meropenem, piperacillin/tazobactam) and fluoroquinolones (levofloxacin). The definition of DTR excluded antimicrobial susceptibility testing results for ceftolozane/tazobactam, imipenem/relebactam and ceftazidime/avibactam.

**Results:**

A total of 3515 Enterobacterales and 786 *P. aeruginosa* isolates were collected in the UK (2020-2024). Overall, 96.6% of the Enterobacterales were susceptible to C/T by the EUCAST breakpoint. Ceftazidime/avibactam, meropenem, ertapenem and amikacin also inhibited >95% of the isolates at their respective EUCAST susceptible breakpoints. C/T was able to inhibit 96.2% of the 339 isolates with the ESBL-positive, non-CRE phenotype; the cephalosporins were inactive versus this phenotype. Among the *P. aeruginosa*, C/T was the second most active agent tested (after colistin), with 97.3% of the isolates interpreted as susceptible. C/T was able to inhibit 78.2%, 80.2%, and 73.2% of the cefepime-, ceftazidime- and piperacillin/tazobactam-resistant isolates, respectively. C/T was also the most active β-lactam agent versus the MDR (75.3% S) and DTR (43.8% S) *P. aeruginosa,* inhibiting approximately 9 and 19 more percentage points than ceftazidime/avibactam.

**Conclusions:**

C/T exhibited good activity against most Enterobacterales, including *E. coli and K. pneumoniae* presumed to carry an ESBL without co-carrying an expressed carbapenemase. As such it could be useful as a carbapenem-sparing therapeutic option for those UK patients with infections caused by ESBL-producing, CRE-negative Enterobacterales. Regarding *P. aeruginosa,* the potent in vitro activity displayed by C/T versus drug-resistant phenotypes suggests it could be a highly effective therapy for patients with infections caused by this organism that don’t respond to standard-of-care antimicrobials.

